# Microbiome pattern of *Lucilia sericata* (Meigen) (Diptera: Calliphoridae) and feeding substrate in the presence of the foodborne pathogen *Salmonella enterica*

**DOI:** 10.1038/s41598-021-94761-w

**Published:** 2021-07-27

**Authors:** Lavinia Iancu, Iulia Roxana Angelescu, Victoria Ioana Paun, Carlos Henríquez-Castillo, Paris Lavin, Cristina Purcarea

**Affiliations:** 1 Department of Microbiology, Institute of Biology Bucharest of Romanian Academy, Splaiul Independentei, 296, 060031 Bucharest, Romania; 2 Department of Criminal Justice, University of North Dakota, Grand Forks, ND 58202 USA; 3 Laboratorio de Fisiología y Genética Marina, Centro de Estudios Avanzados en Zonas Áridas, 1781421 Coquimbo, Chile; 4 Facultad de Ciencias del Mar, Universidad Católica del Norte, 1781421 Coquimbo, Chile; 5 Departamento de Biotecnología, Facultad de Ciencias del Mar y Recursos Biológicos, Universidad de Antofagasta, Antofagasta, Chile; 6 Laboratorio de Complejidad Microbiana y Ecología Funcional, Instituto Antofagasta, Antofagasta, Chile

**Keywords:** Metagenomics, Microbiome

## Abstract

The microbial diversity and quantitative dynamics during the insect’s development stages constitute recently developed putative tools in forensic and medical studies. Meanwhile, little is known on the role of insects in spreading foodborne pathogenic bacteria and on the impact of these pathogens on the overall insects and feeding substrate microbiome composition. Here, we provide the first characterization of the bacterial communities harbored in adult and immature stages of *Lucilia sericata*, one of the first colonizers of decomposed human remains, in the presence of the foodborne pathogen *Salmonella enterica* using 16S rRNA Illumina sequencing and qPCR. The pathogen transmission from the wild adults to the second generation was observed, with a 10^1.25^× quantitative increase. The microbial patterns from both insect and liver samples were not influenced by the artificial introduction of this pathogenic foodborne bacteria, being dominated by Firmicutes and Proteobacteria. Overall, our results provided a first detailed overview of the insect and decomposed substrate microbiome in the presence of a human pathogen, advancing the knowledge on the role of microbes as postmortem interval estimators and the transmission of pathogenic bacteria.

## Introduction

The postmortem interval (PMI) estimation represents a key-step in forensic investigations, in the case of a suspicious death or a homicide by providing the time window in which the death occurred^1^. Most of the time forensic entomology comes to help identify this window, as necrophagous insect species have been used for many years in the court of law as scientific evidence for the PMI estimation^2^, arriving on carcasses minutes after death to feed and breed on the decaying substrate^3–5^. Frequently, the estimation of the time elapsed since death is based on the immature stages rate of development, though, information is also provided by the chronological succession of species/families^6^. Blowflies, including *Lucilia sericata* (Meigen, 1826) (Diptera: Calliphoridae) require this decomposed organic matter substrate for oogenesis and larvae development, as the best survival rates were recorded from such unsterilized mixed bacterial environments^7^. The importance that the study of insect-microbiome interactions has for the PMI estimation relies in the possibility of identifying common and constant insect-associated bacteria and quantifying their transfer in the colonized tissues (such as a human cadaver). In cases where the body is no longer associated with insects, the presence of insect-specific bacteria can suggest that the body had been colonized by insects, and by quantifying these bacteria through the insect life stages, a link with the development stages can be specified, thus, narrowing the estimation of PMI.

Meanwhile, as flies feed and breed, they acquire a part of the microbiome of the living environment^8^. As such, blowflies that feed on decomposed human or animal remains are responsible for transmitting pathogens, most species being vectors for important bacteria, such as *Streptococcus*, *Staphylococcus*, and *Clostridium*^9^. Several studies emphasized the high dispersion ability of blowflies of spreading foodborne pathogens such as various *Salmonella* serotypes^10^, *Escherichia coli*^11^, *Campylobacter* species^12^, and *Helicobacter pylori*^13^. The probability of spreading pathogenic bacteria in a human living environment is very high, given the blowflies synanthropic character^7^. Flies also transfer a part of their own microbiome to their associated environments via regurgitation and defecation^14^. Thus, the human environment can become indirectly contaminated through the activity of flies^15^. While the defecation involves a smaller risk than regurgitation, mainly because the gastro-intestinal tract is a hostile environment for non-specific bacteria, the microbial content from the saliva once eliminated, represents a continuous source of pathogens^16^. However, blowflies can also modify the living substrate microbiome by eliminating substances that have antimicrobial properties, such as defensins and DNase^17,18^.

Earlier studies demonstrated that bacteria are regulating the blowfly attraction and colonization activities^19,20^, as substrates associated with *Proteus mirabilis* were more attractive to flies^4^. At the opposite pole, several bacterial taxa such us *Staphylococcus* or *Bacillus* species^21^ can inhibit the larval growth. In spite of a wealth of information on the vector capacity of flies^9^, very little is known about their role in the environmental bacterial spread, as well as in the bacterial transfer throughout the development cycle of various insect species. As microbes rely on insects for spatial and temporal dispersion, all pathogenic bacteria have many more opportunities to be transferred into the environment given the adults capacity of traveling long distances^7^. Therefore, the microbiome pattern along insect-living environment interactions could lead to a better understanding of the role of flies as main colonizers of decomposed carcasses and as vectors of pathogenic bacteria.

The decomposition process is dependent on many factors, from the cause of death to the environmental factors’ variation, involving both vertebrate and invertebrate scavengers, hosting a poorly understood microbial successional assembly. The decomposing bacterial communities are highly diverse and competitive in terms of acquiring substrate resources^22^. During the last decade, forensic microbiology studies were initiated in search of microbial patterns useful for the PMI estimation^23–25^. These studies targeted the microbiome associated with the decomposition of human remains^26,27^, swine and mouse carcasses^28–30^, different decayed organs^31,32^, and different necrophagous insect species life stages^11,33,34^. Investigations of both cultured and uncultured bacterial communities showed similar dynamics patterns in animal and humans^27^, revealing the importance of using animal models in this field. Several molecular methods of identification were used for the microbiome investigation of different Diptera species, starting with MiSeq sequencing of V4 region^33^, 16S rRNA sequencing^11,35^, 16S rRNA 454 pyrosequencing^36^, and whole shot gun sequencing^13^. All these experimental models helped strengthen the current knowledge on microbes and decomposition. However, these studies focused either on the decomposed tissues necrobiome^22^ or the insects microbiome^33^.

Among blowflies, *L. sericata* is the most common species used in both forensic and medicinal studies, being one of the earliest colonizers of decomposed remains, and a common species used in maggot debridement therapy (MDT)^36^. This blowfly has a cosmopolitan distribution, from North, Central and South America, Africa, Europe, to Australia^37^. Considering these aspects, this species was used as the experimental model in our current research, because the resulted data could be used as reference mean worldwide.

In the current experimental research, *L. sericata* feeding substrate was inoculated with the human pathogen *Salmonella enterica* subsp. *enterica* serovar Typhimurium ATCC 14028. This facultative anaerobic mesophilic bacterium, was reported as highly stable to various stress factors including low pH, osmotic stress, low and high temperatures (2–54 °C), and low water activity, being one of the main foodborne pathogens worldwide^38^. According to a previous study^39^, *Salmonella* caused the highest number of deaths in the USA, as the main human foodborne pathogen costing $2.71 billion for more than one million people. In Europe, over 91,000 cases are reported each year, with involved medical costs of over €3 billion a year, being the second most commonly reported gastrointestinal infection^40^. Most of *Salmonella* serovars were found in the gastrointestinal tract of humans and animals, being eliminated by feces and further transported by insects or other animals to other environments^41^.

In this context, our data aimed to provide novel information on pathogen transmission and bacteria as putative indicators of body colonization by insects, in their absence, by answering specific questions: (a) is *L. sericata* microbiome affected by the presence of a foodborne pathogen in the feeding environment? (b) is the foodborne pathogen transmitted to the new blowfly generation, and if yes, can it be held responsible for future surface/food contamination in a human living environment? (c) what bacteria are associated with the developmental stages of the insect and decomposed liver? and (d) can the fly-associated microbiome provide data for the PMI estimation?

## Results

### Quantitative data on *Salmonella enterica* dynamics in *Lucilia sericata* and liver samples

*L. sericata* adults were collected and fed on swine liver inoculated with low (10^2^ CFU/ml) and high (10^4^ CFU/ml) concentrations of *S. enterica* in order to monitor the pathogen transmission throughout the insect life stages. No *S. enterica* could be identified from the wild *L. sericata* adults collected, nor from the reference specimens that were fed with non-inoculated liver, during the assigned experimental time frame.

**Figure 1:**
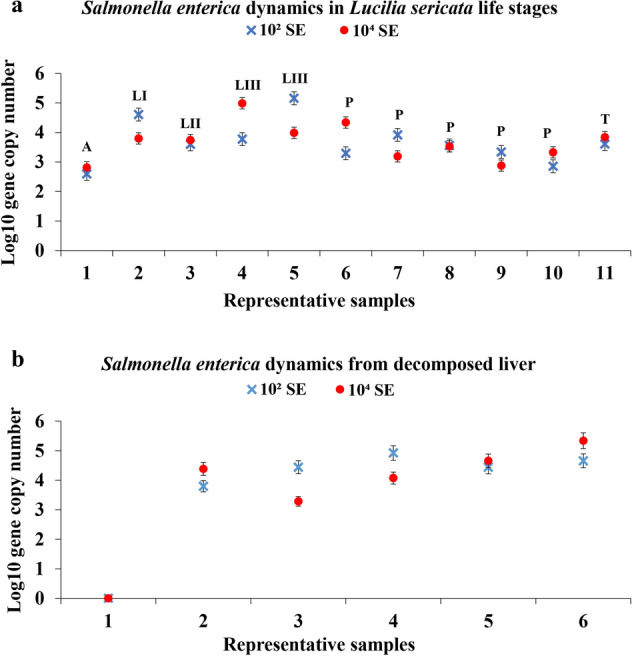
Relative abundance of *Salmonella enterica* throughout (**a**) *Lucilia sericata* life stages (*A* adult, *LI* first instar larvae, *LII* second instar larvae, *LIII* third instar larvae, *P* pupa, *T* teneral specimen) and from (**b**) decomposed liver. Standard deviations are indicated by error bars (*n* = 3).

After being fed for three hours with the inoculated liver, *L. sericata* adults presented similar values for the low (10^2.59^) and high (10^2.81^) *S. enterica* concentrations (Fig. 1a). Following, the abundance of *S. enterica* increased for the first instar larvae (10^3.79^, 10^4.60^), being relatively constant during the second instar larvae stage. The maximum bacterial abundance was recorded for the third instar larvae (10^5.15^), exhibiting 10^2.34^× higher values than the initial adult concentrations. The pupal stage was marked by a slightly descending trend, with maximum and minimum recorded values of 10^4.33^ and 10^2.85^, respectively. *S. enterica* was also identified in the newly emerged adults, with values ranging between 10^3.60^ and 10^3.84^. The bacterial concentration in the new generation was 10^1.03^× higher than the initially assimilated one.

Regarding the differences between the specimens fed with low and high *S. enterica* inoculated liver, the most noticeable difference was recorded for the third instar larvae, with 10^–1.21^× lower concentration in the larvae fed with the low inoculated liver. *S. enterica* content from the low and high initial inoculations was very similar in value between several samples, such as the adult (10^2.59^, 10^2.81^), third instar larvae (10^3.59^, 10^3.74^), pupa (10^3.53^, 10^3.55^) and teneral specimens (10^3.60^, 10^3.84^) (Fig. 1a). Moreover, the specimens fed with both concentrations showed a similar dynamic, namely an ascending trend up to the third larval stage and then a descending slope to the new generation.

No *S. enterica* could be identified in the fresh liver samples, as in the case of the wild *L. sericata* adults. The pathogen content after the first day of inoculation increased up to 10^3.79^ and 10^4.38^ (Fig. 1b). Subsequently the trend was slightly ascendent, reaching a maximum value of 10^5.33^ in the last experimental day. The increase in bacterial abundance of 10^4.65^ (Fig. 1b) corresponded to an increased value in the third larvae stage of 10^5.15^. The liver samples were collected and analyzed as long as the larvae fed on it. Therefore, there are fewer liver sampling points than insect sampling points. Nevertheless, the liver samples may have undergone changes in *S. enterica* concentration due to the actively feeding larvae and the progress of decomposition.

### Bacterial community diversity and profile throughout *Lucilia sericata* life stages and from the liver associated-samples

The bacterial diversity and composition were investigated and found to be specific and clearly delimited for *L. sericata* life stages, represented by the adult, larvae stages, pupa and teneral specimens. Overall, there were 14 phyla, 117 orders, and 274 genera identified for the insect samples, while the liver tissue bacterial diversity contained 28 phyla, 113 orders and 183 genera.

**Figure 2:**
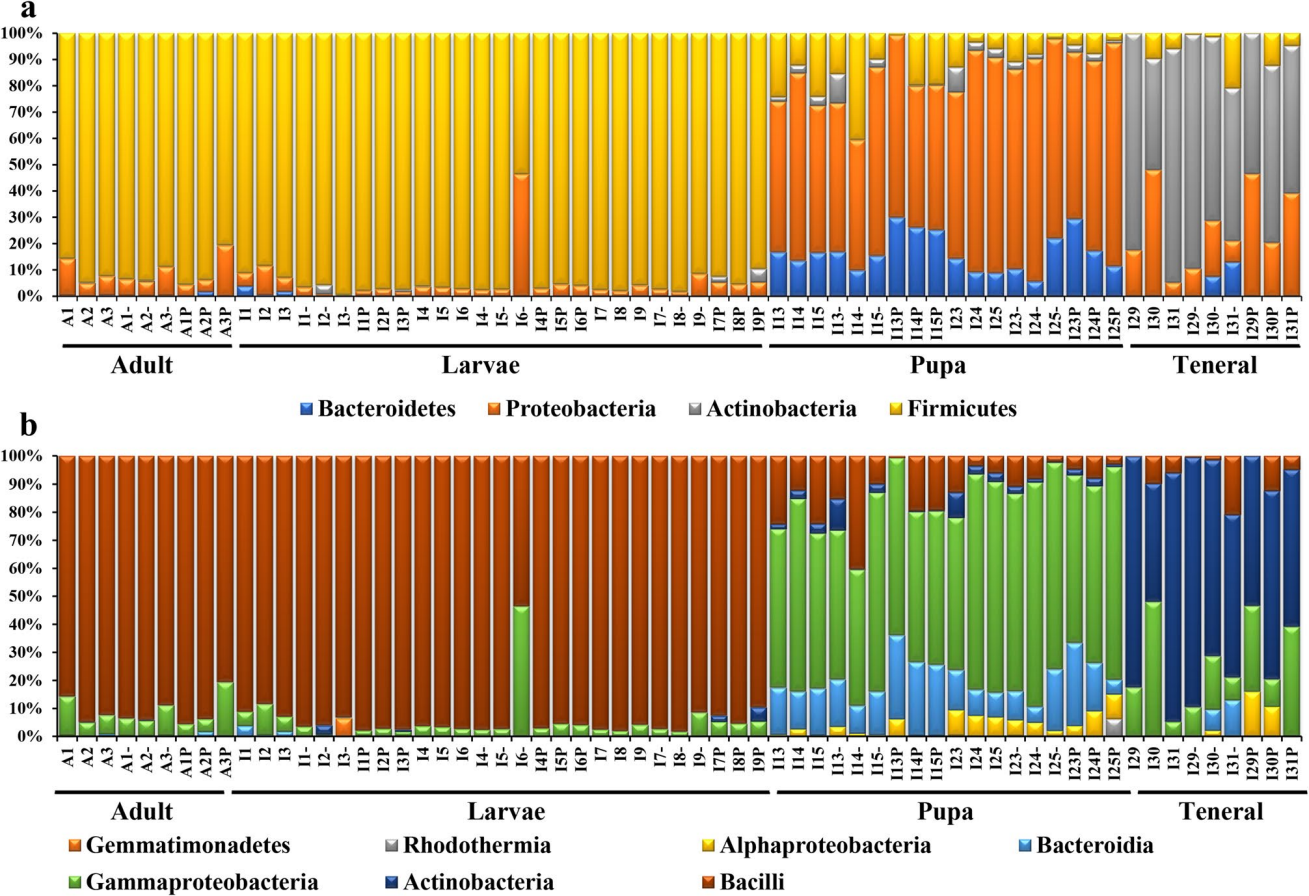
Bacterial diversity composition of *Lucilia sericata* life stages: (**a**) Phylum; (**b**) Class (sample symbols, except control: “–” specimens fed with 10^2^ *S. enterica* concentration; “P” specimens fed with 10^4^ *S. enterica* concentration).

Firmicutes constituted the dominant phyla present in the adult (91.08 ± 5.08%) and larvae (93.47 ± 8.69%) stages, presenting lower relative abundance in the pupa (11.33 ± 10.18%) and teneral (6.17 ± 7.04%) specimens (Fig. 2a). On the other hand, the insect samples presented a greater bacterial diversity during the pupae and teneral stages, with Proteobacteria (67.13 ± 11.29%) prevailing in the pupa, and Actinobacteria (67.51 ± 16.59%) in the teneral specimens, respectively. Bacteroidetes exhibited lower values, being the most abundant in the pupa stage (16.32 ± 7.19%) (Fig. 2a).

**Figure 3:**
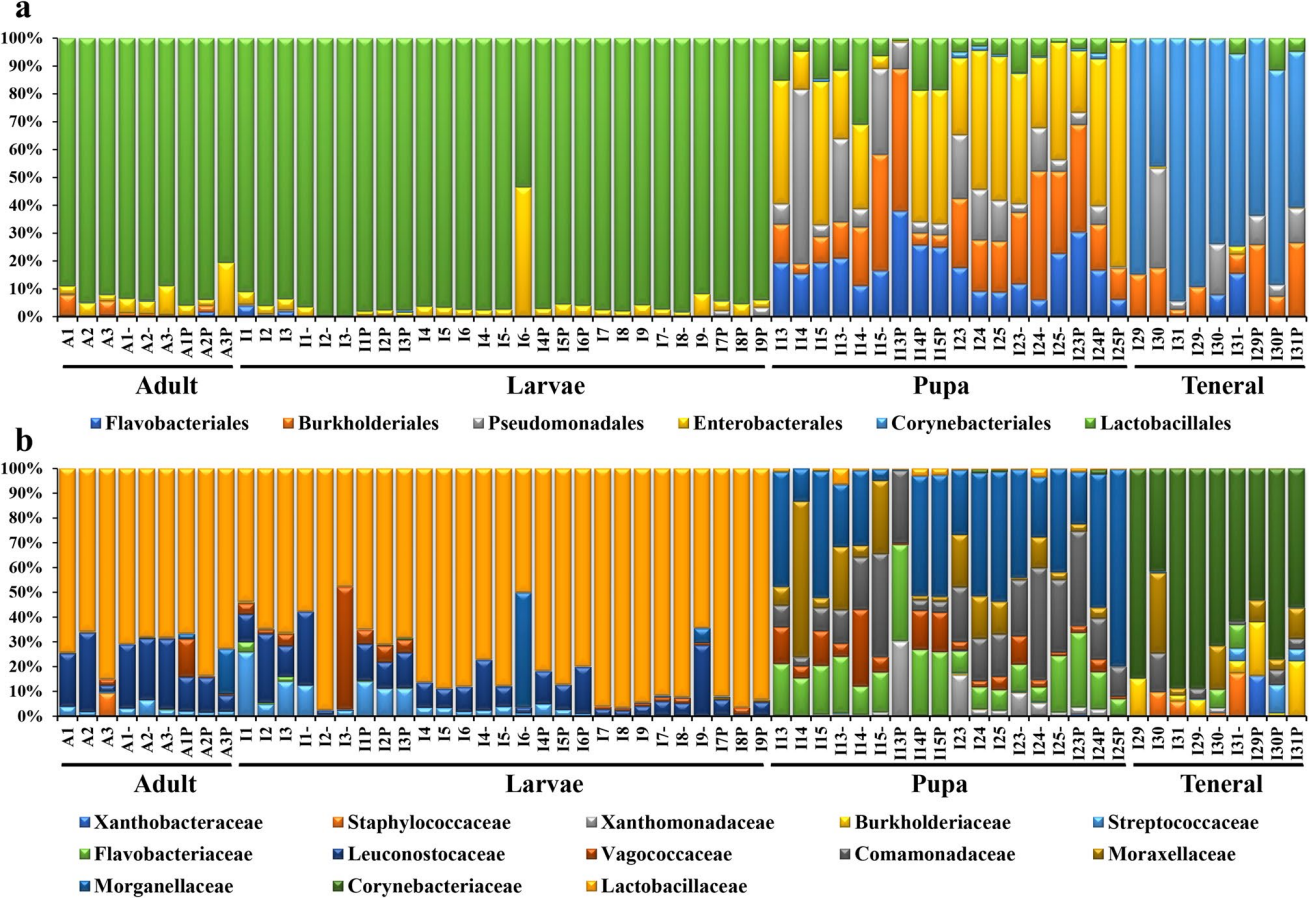
Bacterial diversity composition of *Lucilia sericata* life stages: (**a**) Order; (b) Family (sample symbols, except control: “–” specimens fed with 10^2^ *S. enterica* concentration; “P” specimens fed with 10^4^ *S. enterica* concentration).

Among the most representative classes, Bacilli dominated in the adult and larvae stages (92.81 ± 7.92%), with Lactobacillales as the prevalent order (72.54 ± 14.71%) (Figs. 2b, 3a). The pupa stage corresponded with an increase in the Gammaproteobacteria representatives up to 63.16 ± 9.73% (Fig. 2b), while presenting the highest diversity among the insect samples at the order level (Fig. 3a). Actinobacteria (67.51 ± 16.59%) represented by Corynebacteriales (66 ± 16.04%) prevailed in the teneral specimens (Figs. 2b, 3a).

Out of a total of 155 identified families, Lactobacillaceae and Corynebacteriaceae had the highest relative abundance values (Fig. 3b), while *Lactobacillus* (72.54 ± 14.71%), *Acinetobacter* (20.05 ± 13.96%), *Proteus* (18.63 ± 10.69%) and *Myroides* (16.19 ± 14.05%) were among the most abundant genera detected in all analyzed insect samples.

**Figure 4:**
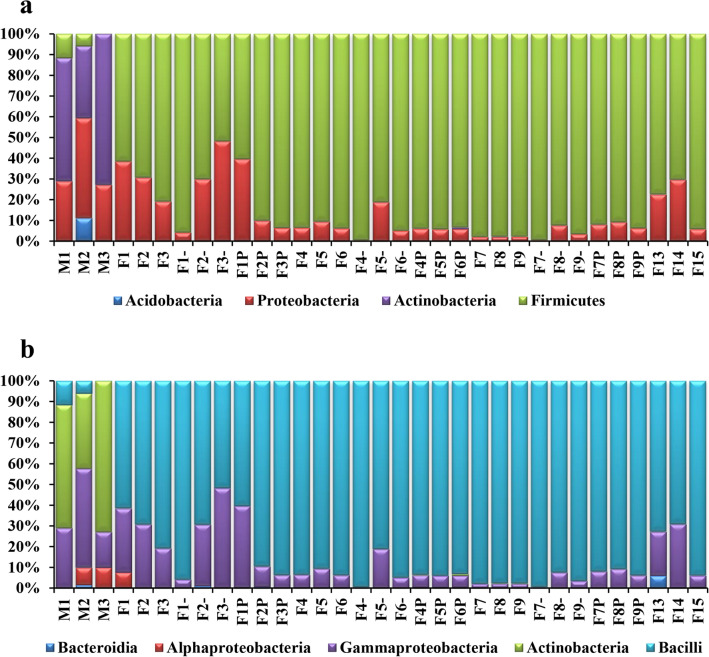
Bacterial diversity composition for the decomposed liver samples: (**a**) Phylum; (**b**) Class (sample symbols, except control: “–” liver inoculated with 10^2^ *S. enterica* concentration; “P” liver inoculated with 10^4^ *S. enterica* concentration).

**Figure 5:**
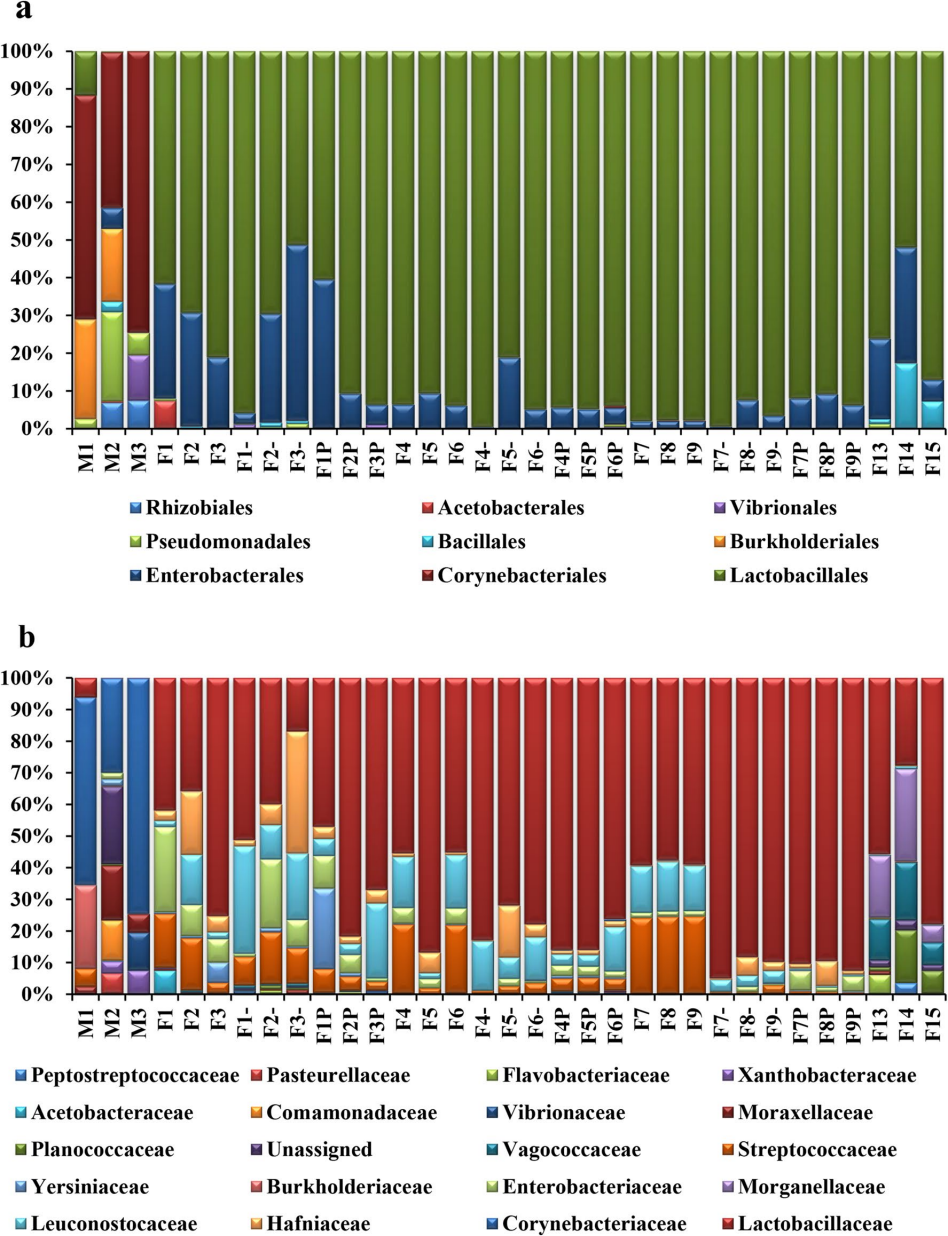
Bacterial diversity composition for the decomposed liver samples: (**a**) Order; (**b**) Family (sample symbols, except control: “–” liver inoculated with 10^2^ *S. enterica* concentration; “P” liver inoculated with 10^4^ *S. enterica* concentration).

No significant differences in the bacterial communities’ diversity were observed in the untreated and treated liver samples with *S. enterica*, while variable community structure was visible during the decomposition process being time dependent (Fig. 4a). The fresh liver samples used as control showed a completely different bacterial community as compared to the decomposed liver tissues (Fig. 4a). While in most samples, Firmicutes constitute the dominant phyla, in the control tissues Actinobacteria had the highest relative abundance (52.81 ± 24.11%), followed by Proteobacteria (30.69 ± 4.75%) (Fig. 4a). During liver decomposition, Firmicutes relative content increased from 83.72 ± 14.70% to 91.32 ± 9.63%, while Proteobacteria registered lower abundances in the first 3 days of decay, recording an average value of 29.09 ± 12.40% (Fig. 4a). A similar pattern was registered at the Order level, Bacilli being the most representative in most samples (86.61 ± 13.45%), except for the control liver tissue (Fig. 4b). Corynebacteriales represented by Corynebacteriaceae (50.86 ± 26.37%) were dominant in the untreated liver, while Lactobacillales represented by Lactobacillaceae (66.84 ± 21.32%) showed the highest relative content in all treated samples (Fig. 5a, b). At the family level, the highest diversity registered during the first experimental days comprised Lactobacillaceae, Enterobacteriaceae, Streptococcaceae, Hafniacea, Leuconostocaceae, i.eg. (Fig. 5b), with *Lactobacillus* (60.94 ± 27.75%) dominating the bacterial community from decomposed liver samples, while *Lawsonella* (50.55 ± 26%) was dominant in the untreated liver.

**Figure 6:**
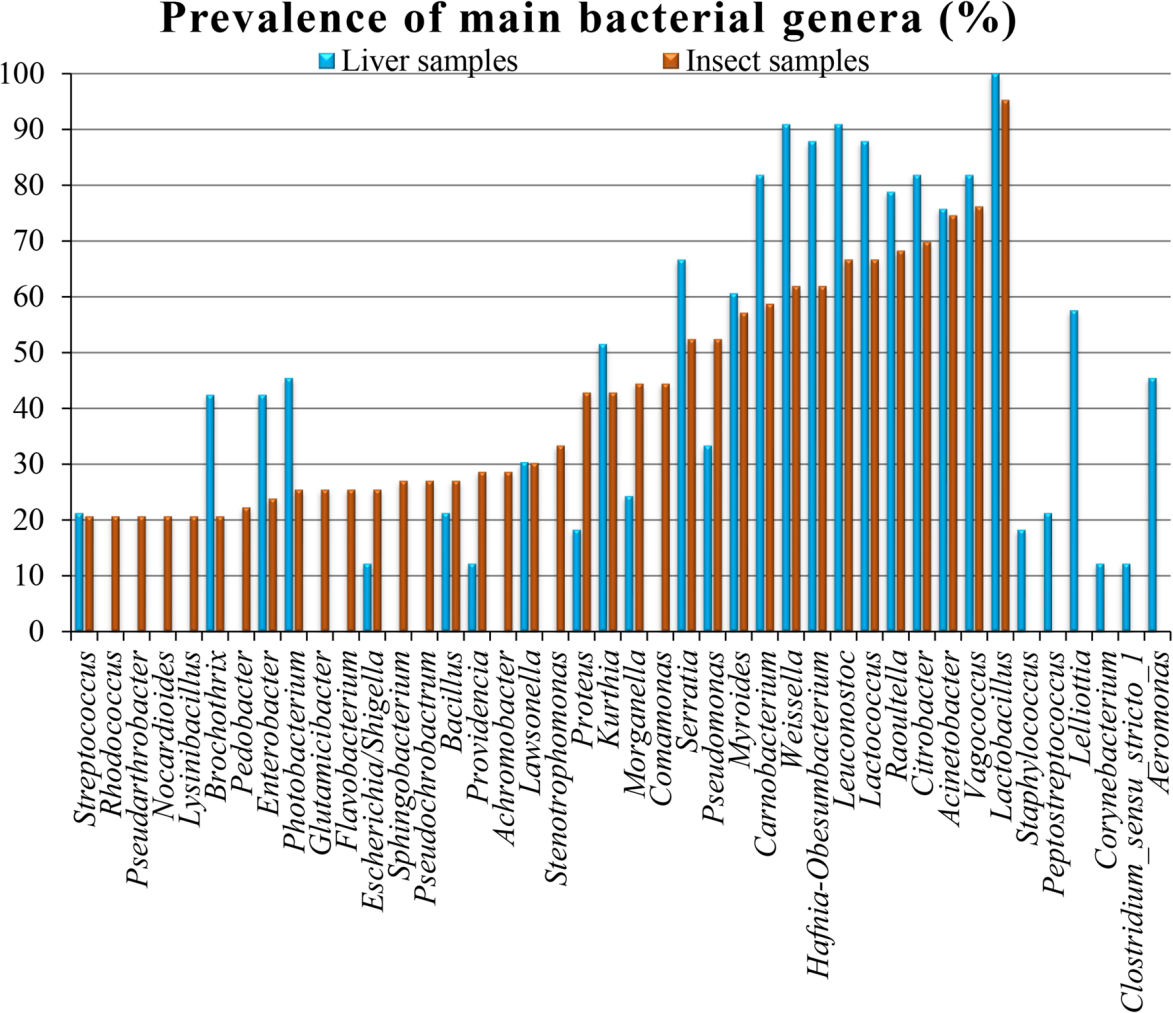
Prevalence of main bacterial genera in insect and liver associated samples. Prevalence was calculated by counting the relative abundance > 0.001% of each individual genus from the total number of samples.

**Figure 7:**
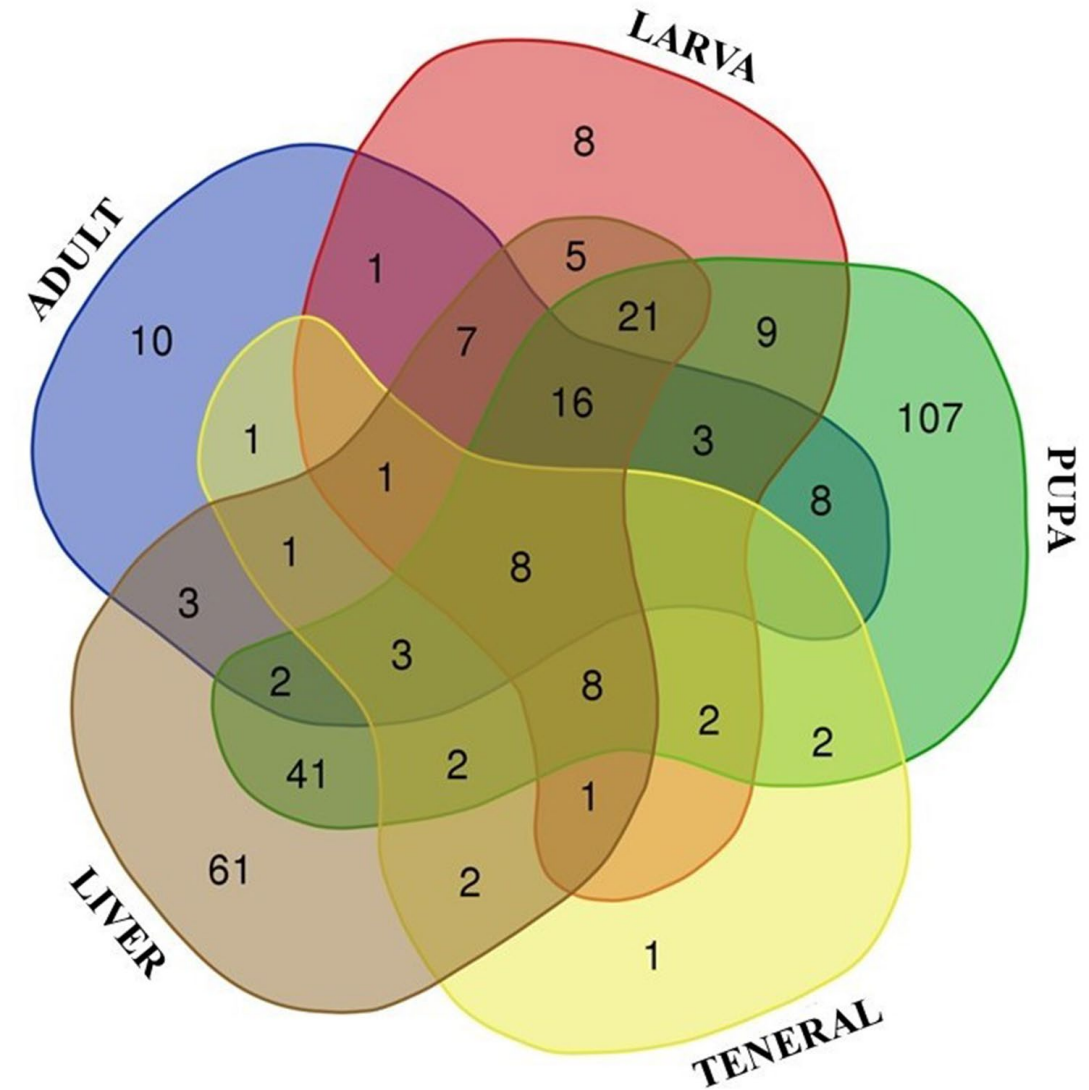
Venn diagram of the bacterial taxa of *L. sericata* and liver tissues. The number of distinct and shared bacterial genera between adult, larva, pupa and teneral insect and liver samples, regardless of *S. enterica* treatment were indicated.

Regarding the genera consistence within the investigated samples, *Lactobacillus* was prevalent in both insect (95.24%) and liver (100%) samples (Fig. 6). Among the other common genera identified (Fig. 7), *Weissella* had different prevalence values, being 29% higher in the liver tissues, representing the second most prevalent genus of this sample type (Fig. 6). Furthermore, *Citrobacter*, *Acinetobacter*, and *Vagococcus* were the among the prevalent genera detected in both insect and liver samples.

Among the total of 457 identified genera, eight were shared by all sample types, from which the most abundant taxa were represented by *Myroides*, *Kurthia*, *Lawsonella*, *Acinetobacter*, and *Lactobacillus*, while the pupa and the liver samples presented the highest number of distinct genera (Fig. 7). When testing for a differential prevalence of bacterial genera, 54 genera were differentially represented between liver and insect samples. Seven genera prevailed in the liver samples, including *Enterobacter*, *Aeromonas*, *Lelliotia*, *Serratia*, *Citrobacter*, *Hafnia Obesumbacterium* and an Enterobacteriaceae, while 47 genera prevailed in insect samples, including *Comamonas*, *Lawsonella*, *Stenotrophomonas*, *Pseudochrobactrum*, *Providencia* and *Morganella* among others*.*

**Figure 8:**
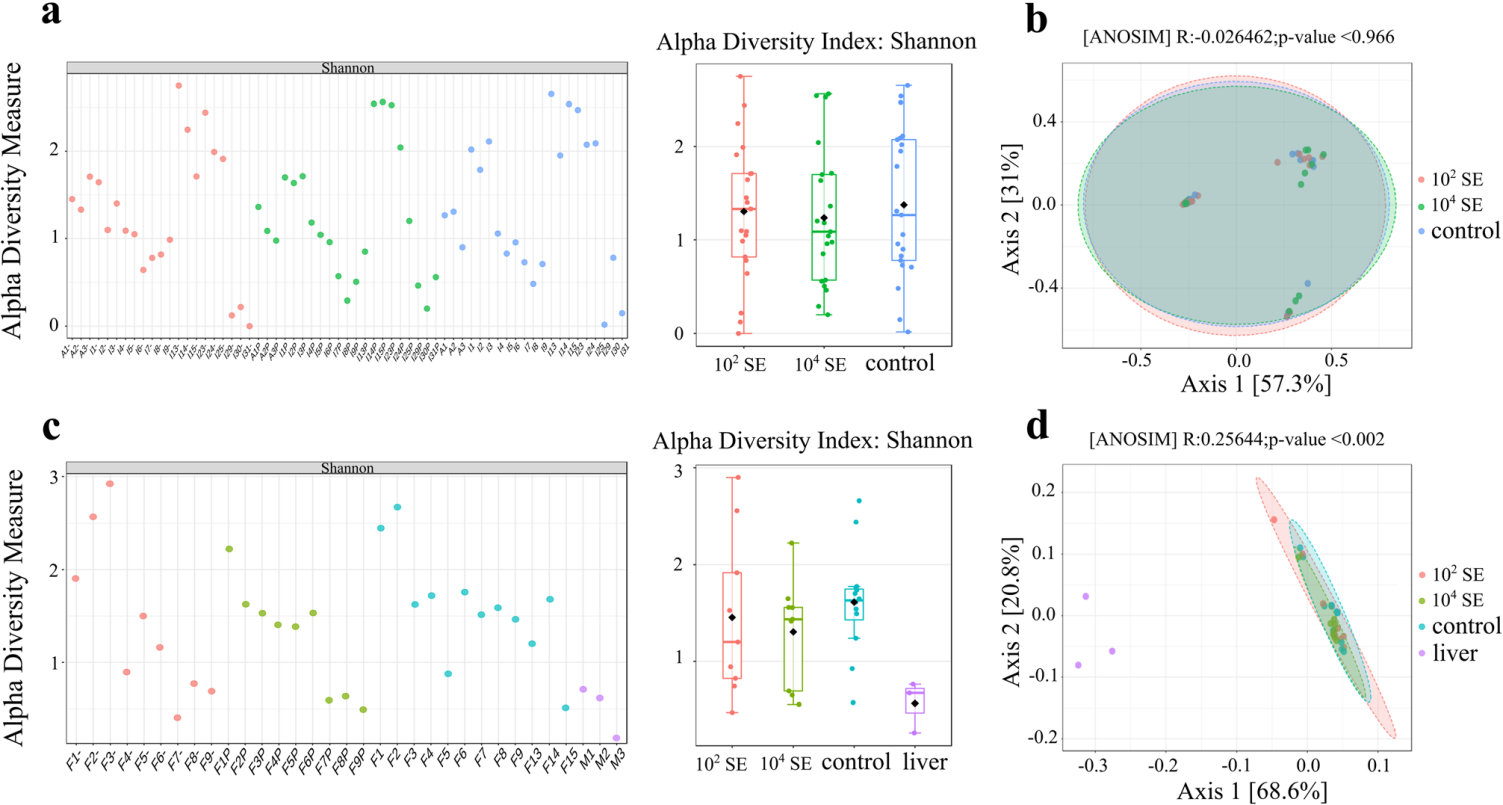
Alpha diversity represented by the Shannon’s diversity index for: (**a**) insect associate samples; and (**b**) liver associated samples. PCoA based on weighted UniFrac distances for (**c**) insect; and (**d**) liver groups.

The Shannon alpha diversity index showed no significant variation among the insect samples subject to *Salmonella* feeding treatment and the samples of insects fed with non-inoculated liver (Fig. 8a). The pupa stage displayed significantly higher values in terms of richness, Shannon index and evenness, while the teneral stage displayed the lowest values of richness and Shannon index (Fig. 8a). Moreover, the presence of *S. enterica* in the feeding substrate did not affect the overall microbiome diversity of the insect specimens, as the two-way ANOVA revealed that the pathogen concentration accounts for < 0.1% of the total variance, while time represented by the development stages (adult, larva, pupa and teneral specimens) accounts for 66.97% (*p* < 0.0001) of the total variance. On the other hand, for the liver samples collected immediately after being purchased the microbiome diversity was the lowest, according to the Shannon index, while the overall diversity observed between treatments showed slight differences (Fig. 8b). The concentration interactions given by the *Salmonella* treatment accounted for 30.08% (*p* = 0.0156), while time accounted for 31.67% (*p* = 0.0127) of the total variance, suggesting that the diversity differences recorded are likely due in part to the pathogens’ presence but also to the decomposition process over time. Furthermore, the alpha diversity for the insect samples had a slightly higher range compared to the liver samples (Fig. 8a,b).

The PCoA based on weighted UniFrac distances was used to further investigate and explain the grouping of samples according to *Salmonella* treatment. In the insect case, the PCoA explained only 88.3% of the variance on the two axes (A1 and A2), where *R* was − 0.026 and *p* = 0.966 (Fig. 8c). The PCoA results were similar for the liver associated samples, where the analysis explained 89.4% of the variance, at an *R* value of 0.256 and *p* = 0.002 (Fig. 8d).

## Discussion

*Salmonella enterica* was introduced in the experimental model to observe whether or not this foodborne pathogenic bacterium can cause changes in the general structure of the microbiome during *L. sericata* development, as well as in the inoculated substrate, and whether or not these quantitative and qualitative changes would affect a possible estimation of the insect development stage. At the same time, the pathogen transfer during fly development was investigated. Given the interaction of blowflies with a large number of bacterial species they are ideal candidates for the study of fly-bacteria interactions. Our study involving *L. sericata* and *S. enterica* helps to understand these interactions in the wild, and their forensic and medical repercussions, by providing data on the bacterial specificity for certain insect life stages that can be used to track their transfer in the colonized tissues. By identifying bacteria-life stages specificity the development stage of the insect along with the body decomposition can be estimated.

For humans, the infectious dose of *Salmonella* strains depends on age, physical condition, health status, as well as the bacterial strains involved in the infection. In general, 10^5^–10^6^ CFU (colony forming unit) bacterial cells are necessary to induce acute salmonellosis^42^. This large inoculum is needed due to gastro-intestinal tract conditions, such as low acidity, presence of bile salts and enzymes, and normal intestinal microbiome which generates nutrient competition. A low gastric acidity related to a variety of medical conditions or in the elderly population can decrease the infectious dose to 10^3^ cells^43^. Consequently, for the current study the feeding environment of *L. sericata* was inoculated with 10^2^ CFU/ml and 10^4^ CFU/ml. An important aspect from a forensic perspective is that there was no registered delay in the colonization of the *Salmonella* inoculated liver by *L. sericata*, indicating that the presence of this pathogen did not prevent the feeding of the adult specimens. A previous study^44^, where pigs were exposed to contamination with *Salmonella enterica* subsp. *enterica* serovar Typhimurium by direct intranasal inoculation, or indirect, by environmental contamination required more than 10^3^ *Salmonella* to induce an acute infection. Moreover, the market-exposed pigs during routine resting or holding could be infected by *Salmonella* when subjected to a bacterial concentration of 10^3^ CFU^45^. As resulted from these previous studies^44,45^, a *Salmonella* concentration of 10^4^× can cause an infection in a weakened animal or human, by food/surface contamination.

Regarding the overall microbiome characterization, studies investigating the fly’s bacterial content showed that Proteobacteria, Firmicutes, Bacteroidetes and Actinobacteria prevailed in both blowfly and housefly life stages^9,13,46^, while another study^36^ revealed that Flavobacteria dominated the pupa stage. Singh and collaborators research^36^ showed that the microbiome pattern was specific and similar for the adult and larva stages, being characterized by Firmicutes with Lactobacillales, for the pupa being dominated by Proteobacteria with the greatest order and family diversity, and for the teneral stage prevailing Actinobacteria with Corynebacteriales. In a recent study, Actinobacteria was not that consistent among *L. sericata* and *L. cuprina* dissected organs, however, were more abundant^46^. Interestingly, during the present research Actinobacteria was present in high abundances in the teneral specimens, suggesting that this phylum can play a role within the species metabolic activities. In contrast, another study revealed that the microbiome between the adult and eggs, and between the pupa and larvae stages was similar^33^. Although a bacterial diversity delimitation, characteristic for certain stages of development could be emphasized, this was not reflected in the diversity dynamics of the liver samples, which were dominated by Firmicutes. Most studies revealed Gammaproteobacteria as the main class identified from the blowfly specimens^9,11,33,35^, with a relative abundance of up to 81%^11^. Furthermore, in Maleki-Ravasan et al.^11^ Morganellaceae represented the most abundant family detected both by culture-dependent and independent methods, while in the current survey Lactobacillaceae dominated, along with Corynebacteriaceae. At genera level, *Citrobacter* was identified among the most prevalent taxa, being also identified by both methods mentioned above from *L. sericata* life stages^11^. This genus plays a role in the production of putrefaction products, such as indole, by converting tryptophan, but also has the ability to ferment lactose^47^, being among the most prevalent genera detected in our samples.

The same recent studies on *L. sericata* bacterial content^11,35^ revealed *Proteus* as the most abundant genus detected, being identified from the digestive tract, salivary glands and the adult stage^48,49^. However, the study of Gasz et al.^46^ revealed the dominant presence of *Pseudomonas* and *Corynebacterium* in all *L. sericata* organs, while *Proteus* was observed in low abundances. During our investigation, this taxon was the 7th most abundant genera, *Lactobacillus* being the most abundant in all investigated samples. The presence in high number of *Lactobacillus* can be explained by this genus fermentative abilities, being also well adapted to the insect hosts^50^. This genus was identified as being more abundant in the insect salivary gland^36^, and was able to inhibit other bacterial growth by producing bacteriocins^51^. However, in the recent study of Gasz et al.^46^, *Lactobacillus* was identified in lower quantities, compared to our study, where this taxon prevailed. The presence of *Lactobacillus* in low abundance^46^ can be a consequence of the investigated organs and feeding substrate that differed from the initial bait (dog dung), the later one could also explain the variation between insect and bait samples. Furthermore, *Proteus* was previously identified as less abundant as 0.4% in *L. sericata* specimens^33^, however, it was demonstrated that this bacterium can transmit swarming signals to attract blowflies^52^, so its presence in insects may be explained by its acquisition from the surrounding environment. Even though in our study Proteus was one of the top ten most abundant genera, it was ranked 16th in prevalence in the insect samples, and 26th in the liver; the highest values being recorded in the last experimental day; 18.86% and 28.75%, respectively. In the insect samples, the higher relative abundance of 37.02% was recorded for the pupa stage, not being detected in the adult, first and second larvae life stages. In addition, *Providencia*, *Vagococcus*, *Lactococcus*, and *Leuconostoc* prevailed as main genera detected in *L. sericata* life stages^33^. *Lactococcus* was previously reported as being associated with *L. sericata*^11,36^, while in our study it was among the top ten genera in the insect and liver samples, constituting a shared taxon for the adult, larva, pupa and liver samples. *Lactococcus* has a homofermentative metabolism^53^, while *Leuconostoc* is a heterofermentative bacteria^54^, their presence being associated with the fermentative phase of decomposition. Furthermore, *Providencia* was previously associated with insects^55^, being considered as an opportunistic human pathogen, similar to *Vagococcus*^56^.

Regarding the investigated life stages, several authors researched the eggs from the first and second generation, third instar larvae, pupa and first-generation adult^33^. Others studied the first and second instars, male and female specimens, and the digestive tract of the third instar larvae^11^, as well as the internal and external contents of the third instar larvae. These type of studies revealed via culture-based bacteriological method that more bacterial species were present in the third instar larvae after feeding, opposite to the unfed specimens, with a three to seven ratio, respectively^11^, but also that the larvae external content presented significant higher Enterobacteria abundances. Simultaneously, the same study showed that several bacteria genera, such as *Enterococcus faecalis* and *Proteus* spp., were present in most of *L. sericata* life stages, having transstadial and transovarial transmission routes^11^. Our data revealed *Lactobacillus* dominated in the adult and larvae stages being present in all investigated life stages.

In terms of pathogen introduction in an experimental model, a study of bacterial transmigration emphasized that the inoculation of mouse bodies with *Staphylococcus aureus* did not affect the overall microbial diversity pattern^22^, not even in organisms that have been sterilized on the surface. However, even if our data showed a similar trend, it is of note that both the inoculated substrate and *L. sericata* life stages that fed on it were investigated, unlike the previous research that limited the access of invertebrate decomposers^22^. This aspect is very important, since it is known that the insect presence can have an influence on the microbial composition of the feeding substrate. Concerning the blowfly’s ability to perform the microbiome exchange in and from the living environment, carrying and transmitting pathogens, was demonstrated by the same microbial patterns identified both in flies and the feces of sympatric animals^9^. Additionally, during the decomposed tissue pre-digestion, blowflies secrete certain enzymes that have antimicrobial actions, thus having the potential to alter the microbial pattern of the substrate^36,57^. These excretions/secretions (ES) with antimicrobial properties can be one of the reasons why many of the bacteria identified in the liver samples were not transferred to the insect specimens. However, the interaction between *S. enterica* and *L. sericata* life stages and liver samples must be considered, as these interactions can play a role in the bacterial acquisition and transmission^58^.

As revealed from the present and past studies, flies can contain numerous pathogens of human and veterinary importance, such as *Enterococcus*, *Streptococcus*, *Pseudomonas*, *Proteus*, and *Serratia*^9^. All of these genera comprise numerous species, that can cause different infections in animals and humans, from minor illnesses to deadly diseases, such as the streptococcal toxic shock syndrome^59^. Some species are present in the gastrointestinal tract of healthy people, such as *Enterococcus* species, but they are also the cause of severe nosocomial infections in hospitalized patients, being inherently resistant to various antibiotics^60^. These infections often target people who are hospitalized or have a compromised immune system, but the infection in healthy individuals can also occur through the consumption of contaminated food or water^61^. *Staphylococcus*, another bacterium with pathological significance, was detected only in the wild adults and teneral specimens with relative abundance values ranging from 9.6 up to 16.18%, data observed in other research where the values were higher in adults than in the larval stage^33^. Among all identified pathogens, *Salmonella* spp. exhibited low abundances of 0.01% in the investigated blowflies and in the initial feeding substrate, assuming that the presence of larvae caused an increase in the substrate abundance of up to 2.01%^36^. Our data could support this statement, given that an increase in the liver bacterial abundance corresponded to an increased *S. enterica* value in the third larvae stage.

Considering all these aspects, the identification of these possible bacterial pathogens in different insect species is very important, in order to monitor their transfer in both invertebrates and humans’ living environments. However, the living environment must be also examined, as most studies investigated only the fly bacterial content, and as has been demonstrated so far^36^, *L. sericata* microbiome is thoroughly connected to the feeding substrate represented by the decomposed organic matter. As such, an increase in abundance of several pathogenic bacteria in the liver samples, identified at the same time in lower abundance in the insect samples, could suggest that the feeding substrate abundances were increased by the feeding larvae^36^. In addition, the experimental introduction of a potential “disturbing” element, as the inoculation of the feeding substrate with *S. enterica*, could bring new and interesting information about the overall microbiome structure. Moreover, by studying the transmission of these pathogens and the environment of the serovars, that can be represented by the gastro-intestinal tract of humans, farm animals, reptiles, birds, and insects, we can have a better understanding on the dispersal of these microorganisms.

Regardless of the acquired pathogens throughout their lifetime, the fly’s microbiome can provide useful data during forensic studies regarding the presence or even the development stage of the insects present on the body, since it was not influenced by the artificial introduction of a specific bacterial taxa. Nevertheless, other pathogens (with increased antibiotic resistance) must be tested to see if their presence alters the microbiome structure during the different development stages of various necrophagous insect species.

## Material and methods

### Experimental setup

**Figure 9:**
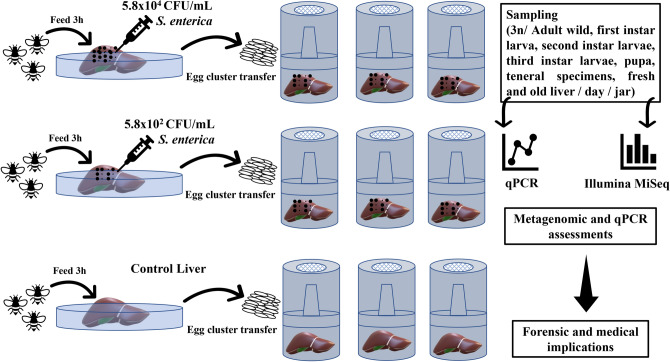
Visual representation of the experimental setup, methods and expected outcomes.

The hypothesis of this study relied on the blowfly’s potential of transmitting pathogens and on their role as main colonizers of decomposed human remains. Thus, the quantitative transmission of *Salmonella enterica* subsp. *enterica* serovar Typhimurium ATCC 14028 through one generation of *L. sericata* life stages (adult to teneral specimens) was investigated, as well as the dynamics within the inoculated feeding substrate (swine liver) via qPCR. Moreover, for an in-depth investigation of the pathogen impact and bacterial putative role as PMI estimators, both insect and liver microbiome were investigated via 16S rRNA gene Illumina MiSeq sequencing (Fig. 9).

### *Lucilia sericata* sampling and laboratory rearing

*Lucilia sericata* adults were collected from an urban area of Bucharest (Romania) (N 44° 26′ 49.349′′ E 26° 2′ 45.352′′) during August 2019. Liver tissue was used as bait and the adult sampling was performed via entomological net. Further, the specimens were immediately transferred to a 46 cm rearing cage (BioQuip Products, Rancho Dominguez, CA, USA), transported to the laboratory and reared under constant laboratory conditions (25 °C ± 1 and 60% relative humidity, LD 13:11) (Fig. 9). The species has been taxonomically identified and genetically confirmed.

Adult specimens were subsequently transferred to nine sterilized rearing jars, 21 × 12 cm (BioQuip Products, Rancho Dominguez, CA, USA). Each rearing jar contained 300 g of swine liver. Three jars contained liver without *S. enterica*, three contained liver inoculated with 10^2^ CFU/ml *S. enterica*, while the last three contained liver inoculated with 10^4^ CFU/ml *S. enterica*. Under these conditions the adult specimens were allowed to feed for three hours and to lay egg clusters. After feeding, they were immediately collected and preserved in 200 µl Tris–EDTA pH 8 (TE) buffer at − 20 °C until further investigations. Under aseptic conditions, using a microbiological safety cabinet, and sterilized rearing jars, the egg clusters (one egg cluster/rearing jar) were transferred on the liver containing the same *S. enterica* concentrations, and monitored under constant laboratory conditions (25 °C ± 1 and 60% relative humidity, LD 13:11) (Fig. 9).

All experimental steps (rearing, transfer, isolation, inoculation, molecular assays) were performed under aseptic conditions to avoid any microbial cross contamination.

### Bacterial cultivation and inoculation

*Salmonella enterica* subsp. *enterica* serovar Typhimurium ATCC 14028 was cultivated in BHI broth (Brain Heart Infusion—Merck, Darmstadt, Germany) at 37 °C in aerobic conditions. Fresh over-night culture was washed two times and resuspended in sterile saline buffer. Dilutions of 10^2^ CFU/ml and 10^4^ CFU/ml were obtained. The bacterial concentrations were obtained by serial dilutions (1/10) of the over-night culture (washed and resuspended in sterile saline buffer) with sterile saline buffer.

Samples of 1 ml of the dilutions were inoculated on the entire surface of each liver and the feeding substrates were incubated for two hours at room temperature for colonization. The liver surface was inoculated by five consecutive times with 200 µl bacterial culture of appropriate concentration (10^2^ or 10^4^) using a sterile cotton swab. The bacterial concentration was measured by CFU counting per ml. From previous experiments and determinations, the concentration of the fresh, over-night culture of *Salmonella enterica* subsp. *enterica* serovar Typhimurium ATCC 14028 was predictable, consequently, the desired inoculants were obtained by appropriate dilutions. To confirm the prospective results, the CFU/ml was determined for each inoculum by spreading 100 µl of serial dilutions onto solid BHI medium (BHI supplemented with 1.5% (w/v) agar), followed by incubation at 37 °C until colonies were observed. To calculate the number of CFU/ml, the number of colony forming units on the countable plate was multiplied by 10 (100 µl to ml) × 10^the dilution of the sample^. The results confirmed the expected bacterial concentrations.

Alternatively, liver treated with saline buffer was used as control. The inoculum dilutant was sterile saline buffer, the same used for the bacterial culture inoculations and final resuspension.

### Sample collection and DNA extraction

Insect and liver samples were collected daily, in triplicate, from all nine jars, and preserved in 200 µl Tris–EDTA pH 8 (TE) buffer at − 20 °C. Prior to DNA extraction, the insect specimens were surface sterilized in 70% ethanol and sterile water for 30 s each. The liver was first sampled 1 h after purchase.

Total DNA was extracted from the whole fly homogenates and liver samples, according to the manufacturer protocol from DNeasy Blood & Tissue Kit (Qiagen, Valencia, CA, USA). The DNA extraction comprised an additional step of cell disruption with 2 mm ZR Bashing Beads (Zymo research, Irvine, CA, USA). The samples were vigorously disrupted at 20 °C, 50 Hz-power, for 12 min via a SpeedMill PLUS Cell Homogenizer (Analitik Jena, Jena, Germany). The DNA purity and concentration were measured with a Biodrop spectrophotometer (Denville Scientific Inc, Holliston, MA, USA).

### *Salmonella enterica* quantitative PCR assays

Control adults and liver samples were tested for the presence of *Salmonella* via PCR amplification and no bacteria could be identified. *Salmonella enterica* quantification from the insect specimens and liver tissues was carried out using Sal_enterF/Sal_enterR (5′-AGCGTACTGGAAAGGGAAAG-3′; 5′-ATACCGCCAATAAAGTTCACAAAG-3′) specific primers^62^. The primers were selected given their capability to detect up to 329 *Salmonella* isolates, belonging to all subspecies of *S. enterica*^62^. For the qPCR assays the total DNA was extracted from the liver and insect samples as previously described.

Thermo Scientific Maxima SYBR Green (1×) Master Mix (ThermoFisher Scientific) reagents were used for the investigation in a total volume of 10 µl, containing 10 µM of each forward and reverse primer, 200 ng DNA template and nuclease free water. All reaction components were thawed and kept on ice, while the entire procedure was performed under a microbiological safety cabinet.

The qPCR amplifications were carried out using a Mastercycler ep gradient S thermocycler PCR (Eppendorf, Wien, Austria) and protocol: initial incubation step of 10 min at 95 °C followed by 40 cycles of 15 s at 95 °C, 30 s at 47 °C, and 30 s at 72 °C. A melting curve analysis was included at the end of each program, being used as amplification control.

Quantification was performed by interpolation in a standard regression curve of cycle threshold (Ct) values generated from samples of known concentration of DNA and plotted as C_t_ versus log_10_ (ng DNA/reaction) and considered when R^2^ > 0.99. Standard curve was generated using corresponding PCR amplified DNA fragments from the known pure culture of *S. enterica*.

The DNA decimal dilutions series used for the standard curve ranged between 5.8 × 10^–2^ and 5.8 × 10^–9^ ng/μl. Samples were run in triplicate, with a Ct threshold value over 35 not considered, while no-template controls were included in all PCR runs.

The gene copy number per reaction was calculated according to Staroscik ^63^: Copy number = (ng DNA × 6.023 × 10^23^)/(length × 1 × 10^9^ × 650), and the plot was performed between the logarithmic scale (base 10) of copy gene number and the sampling points.

### 16S rRNA gene Illumina sequencing and data analysis

The concentration of the total genomic DNA from the liver tissue and insects was measured with Qubit fluorometer (ThermoFisher Scientific). The primer pair 341F/805R^64^ was used for the PCR amplification of the 16S rRNA gene fragments (V3–V4 variable region) (McGill University and Génome Québec Innovation Centre, Canada). The amplicons pooled in equal concentrations were sequenced at the McGill University and Génome Québec Innovation Centre via Illumina MiSeq PE300 sequencing platform.

The samples were analysed using dada2 package (v1.11.3) implemented in R (v3.4.4). Forward and reverse primers were removed using cutadapt (v1.2.1)^65^, then sequences were trimmed to 250 bases and filtered (truncLen = c(250, 250), maxEE = 1, maxN = 0, truncQ = 11, rm.phix = TRUE). Amplicon Sequence Variants (ASVs) were inferred from de-replicated sequences. Chimeras were removed using the “consensus” removal method.

Taxonomic assignment of the ASVs was performed using the Silva v138 16S rRNA database. The community analysis was performed in R environment with the phyloseq and MicrobiomeSeq packages^66^. The sequencing depth varied between 601 and 729.269 sequences per sample with a median of 120,703 sequences. The final dataset consisted of a total of 2798 ASVs from 96 samples. The raw data were deposited in the NCBI SRA Sequence Read Archive under the BioProject: PRJNA646601.

Alpha-diversity was used to evaluate the diversity differences at the ASVs level using Shannon’s diversity index, and significant differences were evaluated using t-tests^67,68^. Principal coordinate analysis (PCoA) based on Bray–Curtis dissimilarities^69^ was used to assess the beta diversity. PERMANOVA and ANOSIM tests were performed in MicrobiomeAnalyst platform^67,68^ to determine statistical differences between a priori groups. Microbial community profiles of the variable V3/V4 region of the 16S rRNA gene sequences and graphical visualization were conducted using MicrobiomeAnalyst^67^ with the parameters for counter filters = 4, prevalence in samples = 20%, and inter-quartile variance filter percentage to remove = 10%. Data were normalized using the Total Sum Scaling (TSS) method^67^. Additional statistical analysis was performed using R version 3.6.3^70^, to investigate statistical differences among treatments using a two-way ANOVA, followed by the Tukey multiple comparison test a posteriori using the package dplyr^71^.

Venn diagram of all identified genera was generated using the Bioinformatics and Evolutionary Genomics online calculator^72^.

## Data Availability

The datasets generated during and/or analyzed during the current study were deposited in the NCBI SRA Sequence Read Archive under the BioProject: PRJNA646601.
